# Autophagy in metabolic syndrome: breaking the wheel by targeting the renin–angiotensin system

**DOI:** 10.1038/s41419-020-2275-9

**Published:** 2020-02-03

**Authors:** Kalhara R. Menikdiwela, Latha Ramalingam, Fahmida Rasha, Shu Wang, Jannette M. Dufour, Nishan S. Kalupahana, Karen K. S. Sunahara, Joilson O. Martins, Naima Moustaid-Moussa

**Affiliations:** 1 Department of Nutritional Sciences, Texas Tech University, Lubbock, TX USA; 2 Obesity Research Institute, Texas Tech University, Lubbock, TX USA; 3 Department of Cell Biology and Biochemistry, Texas Tech University Health Sciences Center, Lubbock, TX USA; 4 Department of Physiology, Faculty of Medicine, University of Peradeniya, Peradeniya, Sri Lanka; 5 Department of Experimental Physiopatholgy, Medical School University of São Paulo, São Paulo, Brazil; 6 Laboratory of Immunoendocrinology, Department of Clinical and Toxicological Analyses, School of Pharmaceutical Sciences of University Sao Paulo (FCF/USP), São Paulo, Brazil

**Keywords:** Autophagy, Obesity

## Abstract

Metabolic syndrome (MetS) is a complex, emerging epidemic which disrupts the metabolic homeostasis of several organs, including liver, heart, pancreas, and adipose tissue. While studies have been conducted in these research areas, the pathogenesis and mechanisms of MetS remain debatable. Lines of evidence show that physiological systems, such as the renin–angiotensin system (RAS) and autophagy play vital regulatory roles in MetS. RAS is a pivotal system known for controlling blood pressure and fluid balance, whereas autophagy is involved in the degradation and recycling of cellular components, including proteins. Although RAS is activated in MetS, the interrelationship between RAS and autophagy varies in glucose homeostatic organs and their cross talk is poorly understood. Interestingly, autophagy is attenuated in the liver during MetS, whereas autophagic activity is induced in adipose tissue during MetS, indicating tissue-specific discordant roles. We discuss in vivo and in vitro studies conducted in metabolic tissues and dissect their tissue-specific effects. Moreover, our review will focus on the molecular mechanisms by which autophagy orchestrates MetS and the ways future treatments could target RAS in order to achieve metabolic homeostasis.

## Facts

- The renin–angiotensin system (RAS) is a pivotal endocrine system classically known for controlling blood pressure and fluid balance.
- Overexpression of RAS (angiotensinogen precursor) in adipose tissue not only increases blood pressure, but also causes glucose intolerance, insulin resistance, adipocyte hypertrophy, and inflammation, yet the involvement of RAS in regulating autophagy in the adipose tissue is not well understood.
- Autophagy is crucial in many tissues, including in adipocyte differentiation and is activated with obesity in adipose tissue. By contrast, autophagy is significantly downregulated in hepatocytes during obesity, indicating discordant tissue-specific roles.

## Open questions

- Is there a link between RAS and autophagy in adipose tissue during the onset of obesity; and does RAS overactivation induce autophagy and disrupt energy metabolism?
- What are tissue-specific molecular mechanisms by which autophagy and RAS differentially mediate metabolic regulations in metabolic syndrome?
- Are there common regulators of RAS and autophagy, which could be used as potential targets for developing future therapies to alleviate metabolic syndrome?
- What other knowledge gaps related to RAS, autophagy, and metabolic syndrome remain to be unraveled?

## Introduction

Metabolic syndrome (MetS) prevalence is increasing around the world and this complex, emerging epidemic afflicts more than one third of American adults^1,2^. The criteria for MetS include abnormalities in three of the following measures: (1) increased waist circumference (≥88 cm for women and ≥102 cm for men); (2) high triglycerides (≥150 mg/dL); (3) high blood pressure (systolic ≥130 mm Hg, or diastolic ≥85 mm Hg, or both); (4) low HDL cholesterol (<40 mg/dL for men and <50 mg/dL for women); and (5) elevated fasting glucose (≥100 mg/dL)^1,3^. MetS is a major risk factor for several chronic diseases^1,4^ and the direct association of MetS with chronic diseases, namely type 2 diabetes (T2D) and cardiovascular diseases, underscores the urgency of identifying effective prevention/treatment options^1,5,6^. Although several studies have been conducted to identify the underlying cause of MetS, due to the syndrome’s complexity, underlying cause is still controversial^7,8^. In this regard, evidence from in vivo and in vitro studies has identified physiological processes such as autophagy and renin–angiotensin system (RAS) as important regulators in MetS^9–14^. Hence, this review highlights new insights and unconventional tasks of the pathway that contribute to MetS development and possible future targets for treatment. Furthermore, our main contribution with this review is to identify the relationship between RAS and autophagy’s influence on glucose homeostasis, in organs and tissues^13–16^ and how manipulation of RAS alters MetS.

## Autophagy and metabolic dysfunction onset

**Fig. 1:**
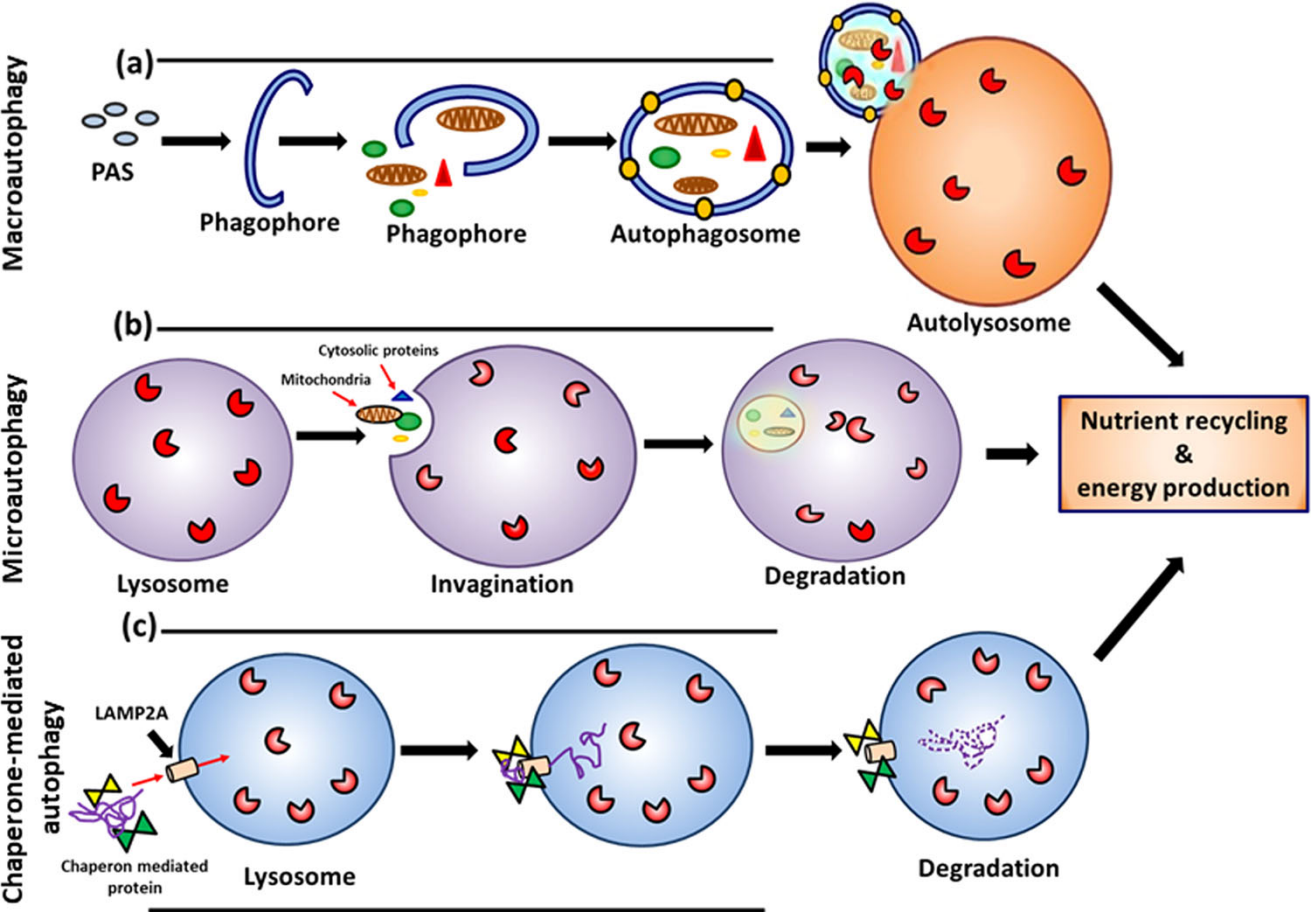
Different types of autophagic processes. **a** Macroautophagy: a process where cytoplasmic content is sequestrated in the double-membrane autophagosome and fused with lysozyme or vacuole for degradation. The process starts with preautophagosomal structure (PAS) which is elongated for matured autophagosome; **b** Microautophagy: a process which is involved in a direct uptake or engulfment of cytoplasmic content into the lysosome for lysis; **c** Chaperone-mediated autophagy: chaperone-dependent cytosolic proteins, along with damaged or malfunctioning proteins, will be guided from cytosol into lysosomes via lysosome-associated membrane protein 2 (LAMP2) for degradation.

**Fig. 2:**
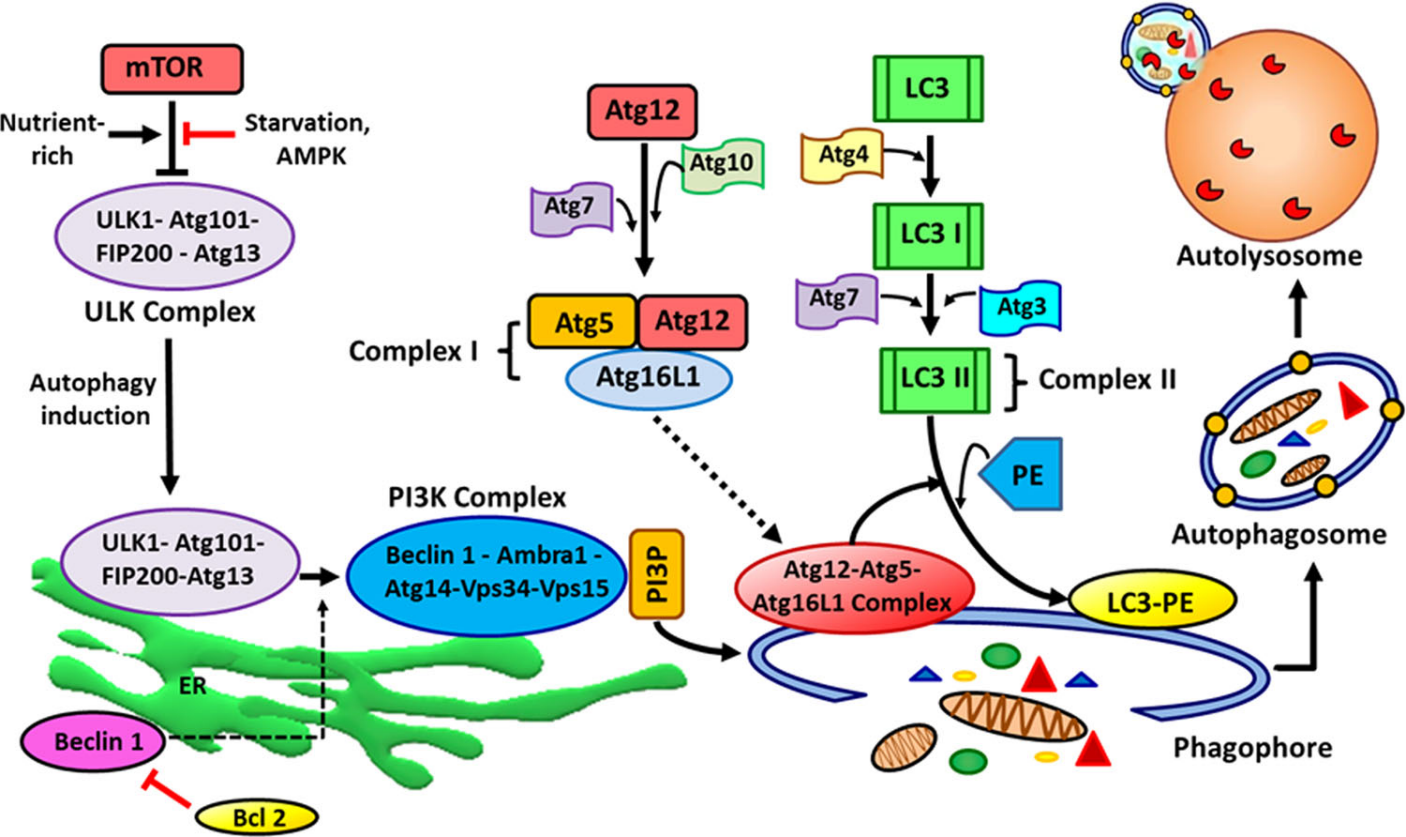
Autophagosome formation and the process of autophagy. Autophagy is initiated by activating the UKL1 complex (consists of ULK1, Atg13, FIP200, and Atg101) and PKI3 complex (consists of Beclin 1, Atg14, Vps34, Vps15, and Ambra1). Phagophores-associated PAS is further elongated by Atg12-Atg5-Atg16L1 complex and LC3-PE complex. After autophagosome formation, it fuses with a lysosome for degradation and recycling of the sequestered content.

Autophagy is a subcellular level process by which cellular components are degraded and recycled in the cell with help of lysosomes or vacuoles^17–19^. This cellular waste-eliminating process plays a pivotal role during starvation and can be stimulated by several factors including nutrient deprivation, reactive oxidative species (ROS)-mediated cell stress, endoplasmic reticulum (ER) stress, DNA damage, accumulation of nonessential proteins, pathogen invasion, and hypoxia^17,20,21^. Autophagy is an imperative process which provides essential nutrients for cell survival. Several types of autophagy—macroautophagy, microautophagy, and chaperone-mediated autophagy—have been recognized with respect to their mode of functions^17,22^ (Fig. 1), but macroautophagy is the most common type found among mammalian cells. Initiation of macroautophagy (autophagy refers to macroautophagy unless otherwise specified) occurs when cytoplasmic content is sequestrated by forming a unique double-membrane structure (autophagosome)^17^, which then fuses with a lysosome to complete the degradation (Fig. 1). There are several autophagy (Atg) genes and protein complexes, such as UNC-like autophagy activating kinase complex (ULK1), phosphatidylinositol 3 kinase (PI3K) complex, Atg12-Atg5-Atg16L1 complex (complex I), and light chain 3- phosphatidylethanolamine (LC3-PE) complex (complex II), that are involved in the autophagic process and are discussed in detail elsewhere^11,17,19,23–26^ (Fig. 2).

Although autophagy is considered as a housekeeping cell process, abnormalities of autophagy have increasingly been associated with metabolic disorders such as obesity^27,28^, insulin resistance^29^, T2D onset^30^, nonalcoholic steatohepatitis^31^, atherosclerosis, and heart disease^32^, which are pathologically linked to autophagy dysfunction and may influence the onset of MetS. Yet the involvement of autophagy in these diseases remains to be properly recognized with additional experiments.

## Autophagy in obesity

Adipose tissue is the primary repository of lipids and also plays a pivotal role in energy metabolism. Adipose differentiation is equipped with extensive remodeling of progenitor cells where removal of cytoplasmic content, specifically mitochondria, is one of the major changes during adipocyte maturation. During early stages of adipose differentiation and adipogenesis, a massive increase in mitochondrial number and mitochondrial proteins is observed^33^. In mature adipocytes, however, mitochondrial count is significantly reduced compared with preadipocytes. This is due to mitophagy (a type of autophagy where the mitochondria is degraded), which is highly activated during maturation of adipocytes and electron microscope results from an early morphological study show active involvement of autophagosomes in removal of mitochondria^34^. Even though mitophagy reduces mitochondrial count during adipose maturation, it is also involved in maintaining appropriate mitochondrial function in mature adipocytes^35^. Although autophagy is crucial for proper functioning and differentiation of adipocytes^9,36,37^, defective regulation during obesity causes metabolic abnormalities, leading to MetS^38–40^.

When autophagy is activated, it facilitates adipocyte differentiation, induces adipogenesis, and enhances fat accumulation in the adipose tissue^37,41^. A clinical study conducted by Kovsan et al. using a range of patients (nonobese, obese, and severely obese with or without diabetes) confirmed a possible link between induced autophagic activity and fat accumulation^27^. The authors showed higher expression of autophagy-related genes (e.g., Atg5, LC3A, and LC3B) in obese patients compared with nonobese patients (autophagy was remarkably induced in obese patients with diabetes). Moreover, autophagic activity was significantly induced in omental fat compared to subcutaneous fat, indicating adipose depot-specific differences^27^. In particular, Kovsan et al. indicated that autophagy is not restricted to differentiation of adipocytes but is also essential for adipose hypertrophy and lipid storage.

**Fig. 3:**
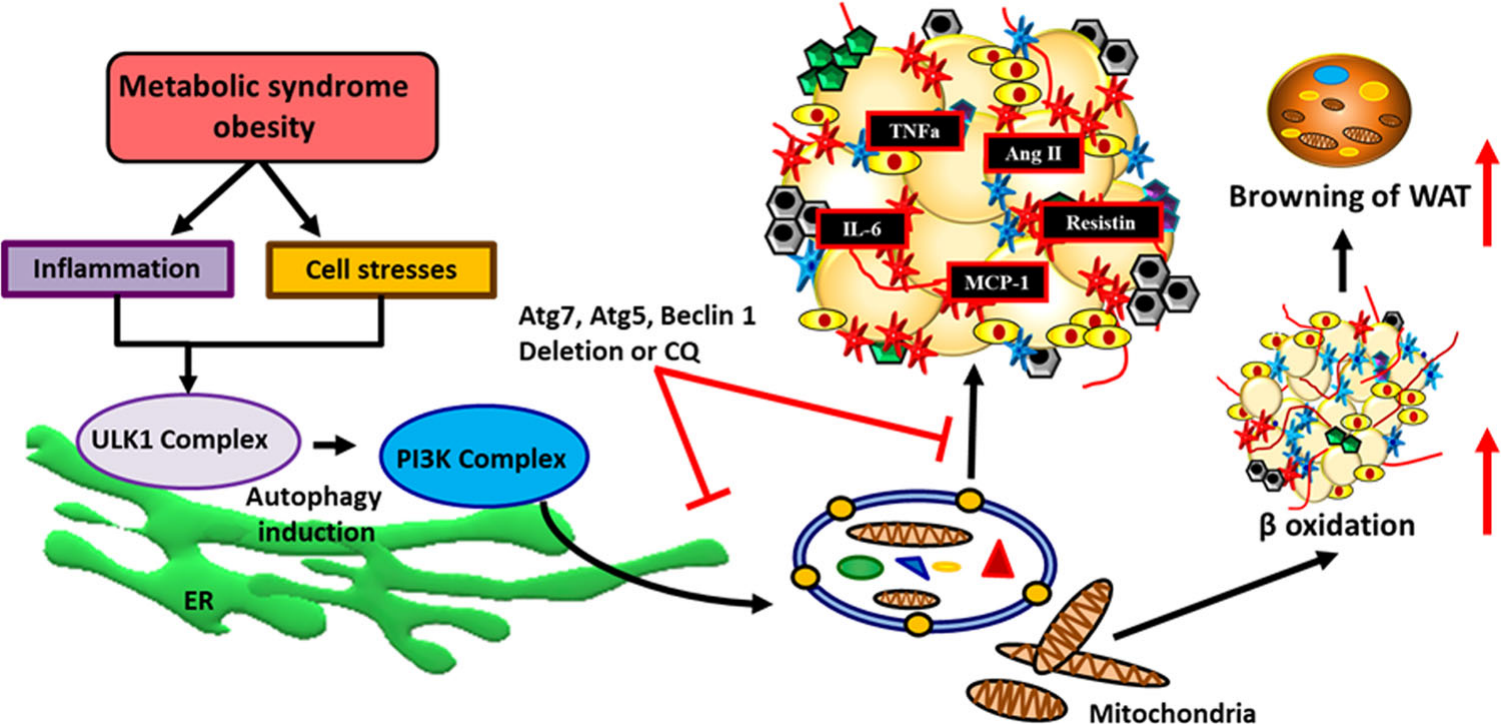
Autophagy and obesity/lipid metabolism. Autophagy plays a pivotal role in adipocyte differentiation and maturation whereas during metabolic syndrome obesity further triggers autophagic activity. Adipose-specific deletion of autophagic genes such as Atg7, Atg5, Beclin 1 and pharmacologic inhibition (chloroquine) of autophagy, reduce excess fat accumulation and induce browning of white adipose tissue.

Gene targeting experiments conducted in different tissues and organs offer a complex picture concerning autophagy and how it is regulated in MetS. Atg7 gene deletion specifically in mice adipose tissue made them resistant to diet-induced obesity. These mice demonstrated lower adiposity and improved insulin sensitivity with higher physical activity compared with control^36,42^. In addition, mice also had increased mitochondrial number, lower triglyceride levels, and lower fat deposition in muscle and liver (liver autophagy is not inhibited in these mice) (Table 1)^36,37^. Moreover, mice with selective deletion of Atg7 gene in adipose tissue had elevated brown adipose tissue (BAT) mass and increased uncoupling protein 1 (UCP-1) gene levels compared with control mice, indicating either higher BAT differentiation or transdifferentiation of white adipose tissue (WAT) into brown-like adipose tissue^37^ (Fig. 3). Similar results were obtained with specific deletion of Atg7 in skeletal muscle to that of adipose tissue^29^ (Table 1). Mice with Atg7 deletion in skeletal muscle had reduced fat mass and were protected against both diet-induced insulin resistance and obesity. However, these mice also showed mitochondrial dysfunction and ER stress (e.g., activating transcription factor 4 (ATF4)) due to loss of autophagic activity^29^ (Table 1). In vitro studies with Atg7 inhibition in murine 3T3-L1 cells confirmed lower protein levels of fatty acid synthase, CCAAT/enhancer binding protein α (C/EBPα), peroxisome proliferator-activated receptor-γ (PPARγ), stearoyl-coenzyme A desaturase 1, and glucose transporter type 4 compared with control^37^. Mice with Atg7 deletion specifically in the hypothalamus showed significantly higher body weight and fat mass, confirming the possible involvement of autophagy in development of an obese phenotype^43^. During prolonged obesity, reduced autophagic activity was observed in both hypothalamus and liver^23,44^, suggesting divergent roles of the autophagic pathway among different organs.

**Table 1:** Effects of autophagy manipulations on metabolism in in vivo studies.

Autophagy manipulation/inhibition	Tissue type	Body weight/adiposity/cell mass	Insulin sensitivity	Insulin secretion	Adipogenesis/adipocyte size/lipid droplets	Cell stress	Lipolysis	Mitochondrial count/deformities	Reference
Atg7 KO mice	Adipose	−	+	NA	−	NR	+	+	^36^
	Adipose	−	+	NA	−	NR	NC	+	^37^
	Liver	NC	NR	NA	+	+	−	+	^57^
	Liver	−	NR	NA	+	NR	NR	+	^56^
	Liver	NC	−	NA	+	+	−	NR	^55^
	Muscle	−	+	NA	−	+	+	+	^29^
	Whole body	−	NR	NA	NR	NR	NR	NR	^56^
	Pancreas	NC	−	−	NR	NR	NR	+	^79^
	Pancreas	−	NR	−	NR	NR	NR	+	^80^
	Pancreas	−	NR	−	NR	+	NR	NR	^81^
Atg5 KO mice	Whole body	−	NR	NR	−	NR	NR	NR	^10^
	Whole body	−	NR	NR	NR	NR	NR	NR	^45^
Chloroquine (CQ) treated mice	Whole body	−	+	NA	−	NR	NR	NR	^10^
	Muscle	−	+	NA	NR	NR	NR	NR	^10^
	Adipose	−	+	NA	−	NR	NR	NR	^10^
	Pancreas	−	+	+	NR	NR	NR	NR	^10^
Atg5 Tg Overexpression	Whole body	−	+	NR	NR	−	NR	NR	^164^

Table provides information about how autophagy manipulation affects metabolism in vivo.

*NA* not applicable, *NC* no change, *NR* not recorded, *−* downregulated, *+* upregulated.

**Fig. 4:**
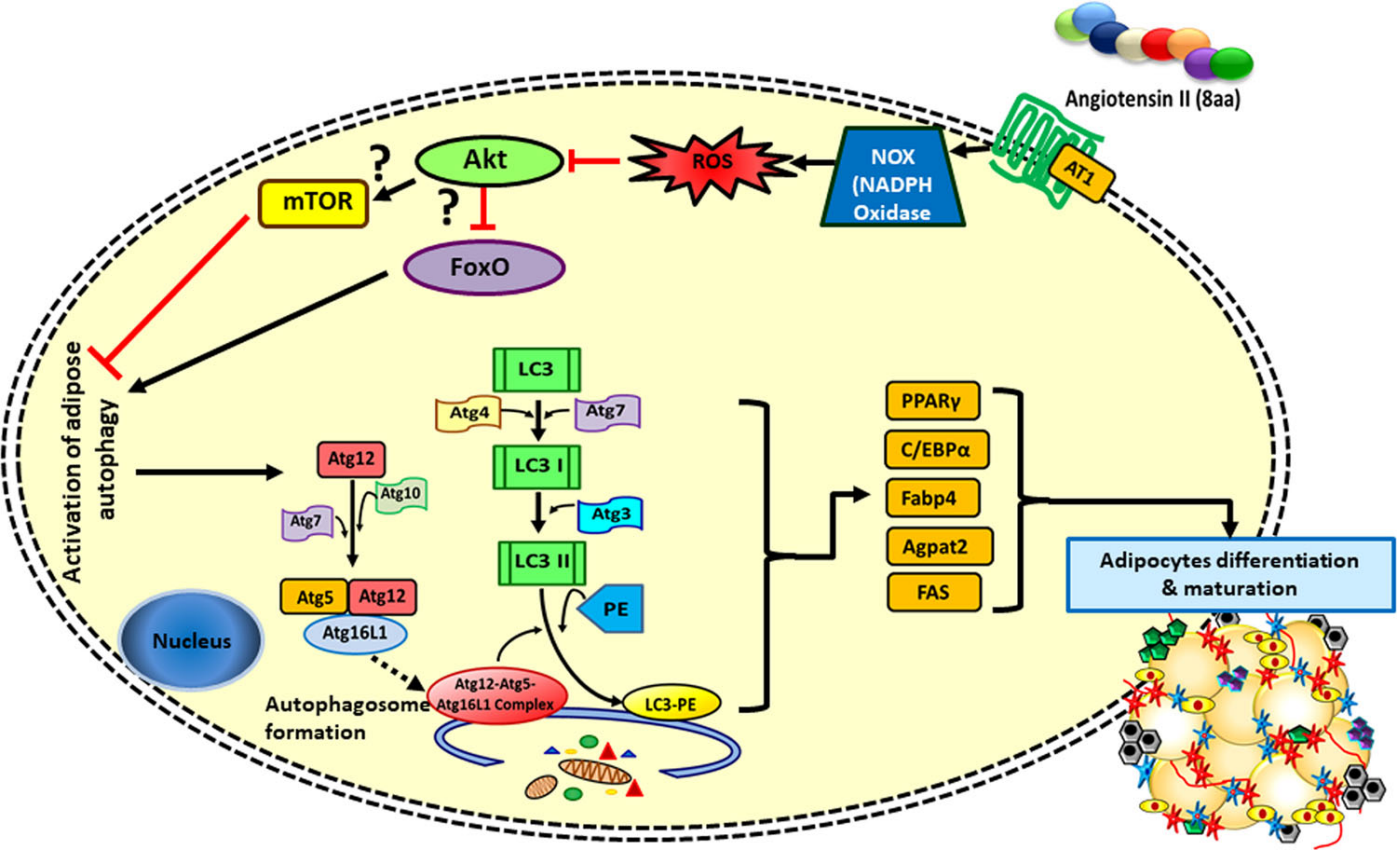
Involvement of autophagy in adipose differentiation. Activated adipose autophagy upregulates the makers (e.g., C/EBP, Fabp4, Agpat2, FAS) of adipose differentiation and maturation. Activation of autophagy could possibly be via angiotensin II-mediated NADPH oxidase and ROS production as well as other triggers such as cell stresses, inflammation, and macrophage infiltration.

Baerga et al. inhibited autophagy by targeted deletion of another autophagy gene, Atg5 (Atg5^−/−^ in mouse embryonic fibroblasts (MEFs)). These MEFs, neonatal pups, and late-stage embryos showed defects in adipogenesis and adipocyte differentiation^9^ (Table 2). After Atg5 deletion, several genes involved in adipocyte differentiation were downregulated; these included *PPARγ*, *C/EBPα*, glycerol-3-phosphate acyltransferase 1, mitochondrial (*Gpam*), fatty acid-binding protein 4 also known as aP2 (*Fabp4*), 1-acylglycerol-3-phosphate O-acyltransferase 2 (*Agpat2*), and perilipin^9^ (Fig. 4). Another mouse study with ubiquitous Atg5 knockout reported lower birth weight, and plasma concentrations of branched-chain and essential amino acids in homozygous Atg5^−/−^ compared to control group. Furthermore, all the homozygous mice died within 1 day post birth due to suckling defect (nutrient deficiency), indicating the importance of Atg5 for early development (postnatal ontogenesis)^45^ (Table 1). Another animal study conducted with autophagy inhibitor chloroquine and targeted disruption of Atg5 gene unveiled possible mechanisms underlining the role of autophagy in obesity^10^ (Figs. 3 and 4) (Table 1).

**Table 2:** Effects of autophagy manipulations on metabolism in in vitro studies.

Autophagy manipulation	Cell type	Adipogenesis/adipocyte size/lipid droplets	Cell stress	Lipolysis	Mitochondrial count	Reference
Atg7 knockdown	Adipocytes	−	NR	NC	+	^36^
	Hepatocytes	+	+	−	NR	^57^
	Mice embryonic fibroblasts	NR	+	NR	NR	^57^
Atg5 knockdown	Mice embryonic fibroblasts	−	NR	NR	NR	^9,10,61^
	Adipocytes	−	NR	+	NR	^37^
Chloroquine (CQ) treatment	Adipocytes	−	NR	NR	NR	^10^
3-methyladenine Treatment	Hepatocytes	+	NR	−	NR	^55^
	Adipocytes	−	NR	NR	NR	^37^

Table provides information about how autophagy manipulation affects metabolism in vitro.

*NA* not applicable; *NC* no change; *NR* not recorded; − downregulated; + upregulated.

Both in vivo and in vitro results confirm that autophagy inhibition reduces PPARγ activity and directly attenuates adipocyte differentiation^10^. PPARγ is a rate limiting enzyme responsible for adipogenesis and fat accumulation in the obese adipose tissue^46,47^. Thus, activation of PPARγ by autophagy can be a mechanism by which autophagy induces obesity^48–50^ (Fig. 4) and it could be a feasible target to prevent autophagy associated with obesity during MetS. Furthermore, activation of PPARγ during obesity may also depend on other factors: e.g., polyunsaturated fatty acids and prostaglandins (e.g., prostaglandin J_2_, prostaglandin D_2_)^51,52^. Therefore, additional research is required to fill knowledge gaps and determine whether the activated PPARγ pathway induces autophagy or vice versa during obesity.

### Hepatic autophagy during obesity

**Fig. 5:**
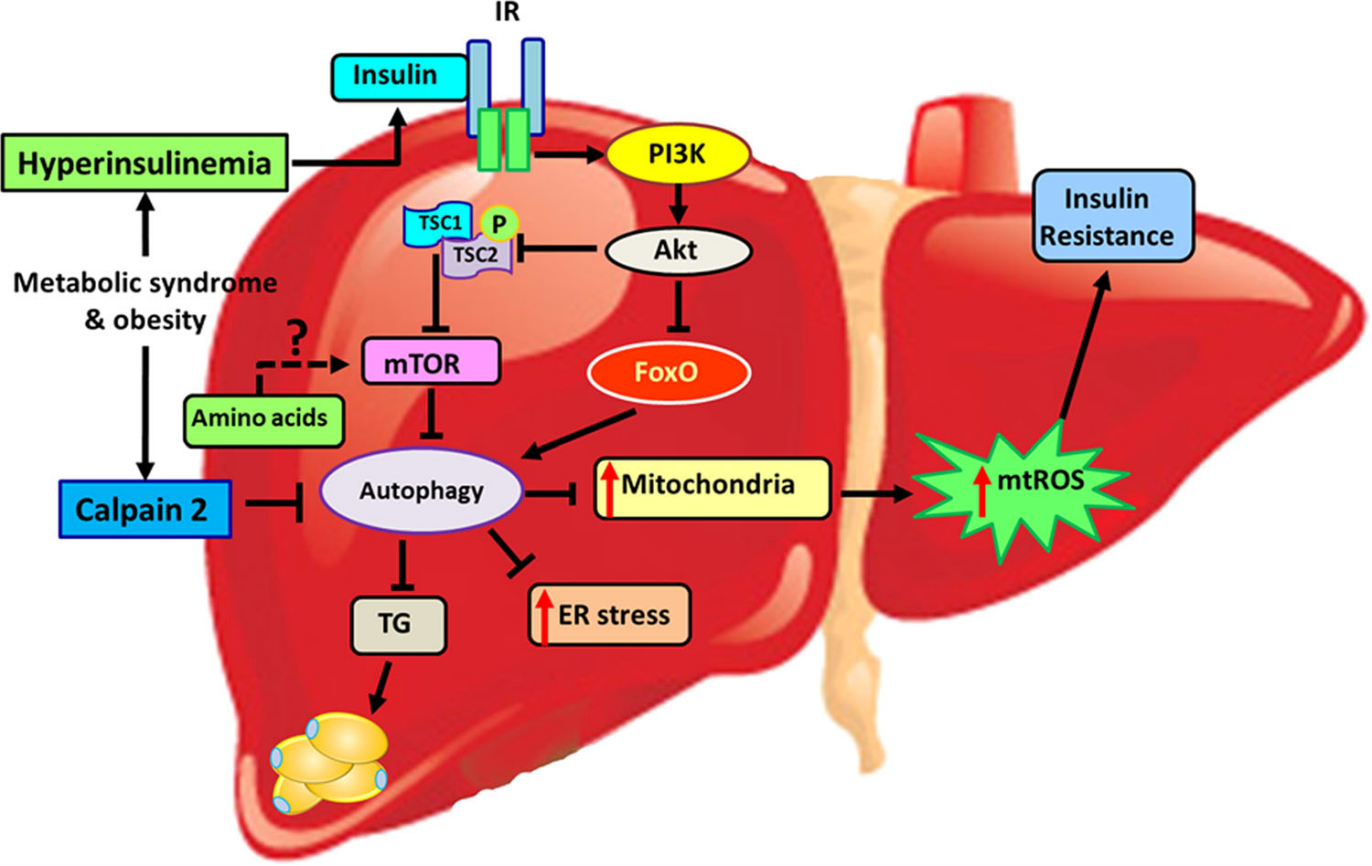
The role of autophagy in the liver during metabolic syndrome. Autophagic activity is significantly reduced in hepatocytes during obesity. Inhibition of autophagy promotes hepatocyte lipid accumulation, malfunction of mitochondria, and cell stresses including ER stress. This, in turn, induces insulin resistance, results in a fatty liver and disrupts the normal liver functions. Induced insulin secretion due to metabolic syndrome will directly affect the autophagy process by activating mTOR and inhibiting the FoxO via PI3K/Akt pathway. Moreover, increased calpain 2 will further inhibit autophagy and increase amino acid concentrations which may contribute to upregulation of mTOR activity during obesity and metabolic syndrome.

The role of autophagy in the liver is surprisingly different from adipose tissue in MetS. During obesity, autophagy is significantly reduced in hepatocytes^53,54^ and impaired metabolism along with deformed mitochondria are observed in the liver^23^ (Fig. 5). In contrast to adipose tissue, inhibition of autophagy promotes hepatocyte lipid accumulation by lipolysis of triglyceride-accumulated lipid droplets^55^ (Fig. 5) (Table 1). Mice with hepatocyte-specific Atg7 deletion, develop fat droplets similar to those observed in fatty liver^56^ (Table 2). However, restoring Atg7 expression was beneficial, as it improved hepatic function with lower ER stress and improved insulin sensitivity^57^. Yang et al. have shown lower protein levels of Atg7, Beclin 1 (Atg6), LC3, Atg5, and elevated p62 (a polyubiquitin-binding protein and accumulation of p62 indicates reduced autophagic activity) in livers of obese mice. In addition, higher ER stress and insulin resistance were observed in these mice due to mitigated autophagic activity in hepatocytes (Fig. 5). Furthermore, reduced hepatic autophagy is reported in both diet-induced and genetic obese models^57^, which could be explained by obesity-associated hyperinsulinemia (insulin inhibits autophagy). Yet, insulin may not be the primary cause for reduced hepatic autophagy in obesity and other possible mechanisms might coexist. One mechanism is through Ca^2+^-dependent protease (calpain 2) where higher levels of calpain 2 in hepatocytes reduce autophagy in obese models^57–59^ and inhibition of calpain 2 increases autophagy^57^ (Fig. 5). Another possible mechanism by which autophagy is reduced in the liver is through forkhead box O (FoxO) transcriptional factor^23^ (Fig. 5). FoxO acts as a key regulator of Vps34 and Atg12, which are responsible for autophagy initiation^23^. Elevated insulin levels and activated Akt suppress FoxO activity, thereby reducing autophagic activity in MetS^23,60^. A recent study conducted in mice shows that melanocortin 3 receptor (MC3R) regulates hepatic autophagy in obesity by possibly affecting transcription factor EB signaling^61^. Thus, long-term inhibition of autophagy due to insulin resistance and hyperinsulinemia in MetS could be explained by reduced FoxO activity in hepatocytes^23^ (Fig. 5). By contrast, some studies conducted in liver of mice demonstrated an induced activity of autophagic markers in liver during obesity^62,63^. These studies show that high fat diet (HFD)-induced hepatic steatosis and obesity-associated ER stress substantially activate autophagy as a protective mechanism against cellular damage^57,63^. Autophagy shields hepatocytes from lipotoxicity-associated ER stress as well as from saturated fatty acid (palmitic acid)-induced apoptosis^63,64^ and this could be why we see an induced autophagic activity during early stages of obesity. Nonetheless, research has further shown that HFD-induced autophagy effects last for first few weeks and autophagic activity is eventually declined due to continued cellular stress in chronic obesity^57,65^. Yang et al. demonstrated that autophagic activity remained effective for 7 weeks of HFD, and it decreased at 16 weeks and was completely eradicated by 22 weeks^57^. Moreover, mRNA and protein levels of Beclin‐1 and LC3 were significantly higher in ob/ob mice (mutant mouse model with severe obesity) compared with wild-type littermates. Interestingly, the same ob/ob mice showed significantly reduced LC3-II level and LC3‐II/LC3‐I ratio compared with wild‐type mice indicating impairments in autophagic process^66^. Although, some of these results show increased expression of autophagy markers and autophagosome number in obesity, without appropriately measuring autophagic flux (total process of autophagosome synthesis, substrate delivery, and lysosomal degradation), we cannot really claim these as results of increased autophagic activity. Hence, additional research is required to clearly understand the cross talk between obesity and autophagy in the liver.

## Autophagy and insulin resistance

Insulin is a known inhibitor of autophagy, and increased insulin secretion observed in obesity has a negative regulatory effect on autophagy^57,67^. In vitro, animal and clinical studies have reported a link between autophagy and insulin resistance, which contributes to MetS. Inhibition of Atg5 and Atg7 in WAT improved insulin sensitivity, glucose clearance^10,36,37^ (Table 1), along with improved insulin signaling (higher phosphorylated (p)-Akt and p–glycogen synthase kinase 3 (p–GSK-3))^37^. However, when autophagic genes Atg3 and Atg16L1 were deleted in mature adipocytes, these knockout mice exhibited insulin resistance compared to control mice. Atg3 and Atg16L1 knockout mice showed impairments in glucose tolerance and insulin sensitivity with increased serum insulin levels indicating development of peripheral insulin resistance^35^. In addition, Atg3 and Atg16L1 knockout mice exhibited dysfunctional mitochondria and increased accumulation of lipid peroxides in adipose tissue depots indicating potential long-term threats of deletion of autophagy in mature adipocytes^35^. Similarly, when autophagy genes were suppressed in the liver, insulin signals were disrupted^57^. Data from patients suffering from obesity and T2D showed reduced activity of mammalian target of rapamycin (mTOR), and lower insulin receptor substrate 1 (IRS1) levels in adipocytes from subcutaneous fat with higher autophagy activation compared with nondiabetic patients^39^. However, the higher number of autophagosomes in diabetic adipocytes could indicate a protective cellular mechanism or a sign of disease progression^39^.

The relationship of autophagy with insulin resistance is possibly mediated via mTOR. mTOR complex 1 (mTORC1) which is activated by insulin and insulin-like growth factor 1 (IGF1), regulates autophagy via several pathways^68,69^, such as (a) PI3K/Akt pathway mediates the inhibition of tumor suppressor complex (TSC1/TSC2) by phosphorylating TSC2, which is a negative upstream regulator of mTORC1. This, in turn, disrupts association of TSC1/TSC2 complex, thereby activating mTORC1 and inhibits autophagy^70–73^. (b) MAPK/ERK also mediates inhibition of TSC1/TSC2 complex and activates mTORC1 to suppress autophagy; this is accomplished by inhibiting ULK complex in mammals or Atg1 complex in yeast by phosphorylating the components ULK1, ATG13, and FIP200^74,75^. (c) In contrast, AMP-activated protein kinase (AMPK) activates autophagy by phosphorylating regulatory-associated protein of mTOR, which ultimately reduces mTORC1 activity^76^. However, in energy-dense situations, mTORC1 phosphorylates Ser_758_ of ULK1 and prevents the interaction between ULK1 and AMPK (AMPK directly phosphorylates ULK1 to activate autophagy especially during lower cellular energy levels), thereby preventing activation of autophagy^11,75,77^. (d) Finally, mTORC1 also suppresses autophagy by phosphorylating other factors, such Vps34 and Ambra1^78^.

During early stages of MetS, autophagy is upregulated in β-cells, increasing pancreatic insulin secretion^11,79^. Inhibition of autophagy in β-cells disrupts cellular equilibrium in the pancreas similar to liver and muscle. Mice with pancreatic β-cell-specific Atg7 deletion had reduced β-cell mass, impaired insulin secretion, and reduced PI3K levels^80,81^ (Table 1). During hyperinsulinemia (autophagy-mediated higher insulin secretion from β-cells) with MetS, exacerbated hepatic mTORC1 activity suppresses autophagy in the liver^57^. Reduced liver autophagic activity, along with lipid accumulation, poses a risk factor for several health conditions including insulin resistance and nonalcoholic fatty liver disease^31^. Concurrently, activated hepatic mTORC1 further phosphorylates the insulin receptor (IR) through ribosomal protein S6 kinase (S6K1), where this overactivation of IR leads to insulin resistance^74,82^. Hence, in the long run, increased secretion of insulin by β-cells decreases hepatic autophagy (causing impaired liver metabolism) and induces insulin resistance in hepatocytes. In addition, inflammatory cytokines also activate mTOR via toll-like receptor (TLR)-mediated PI3K/Akt pathways to reduce autophagic activity during MetS. Although the exact mechanism is poorly understood, Atg7 deletion in β-cells can potentially reduce ER stress-associated unfolded protein responses (UPRs) by reducing PI3K levels (PI3K binds with X-box binding protein 1 to stimulate UPRs in the presence of insulin). Interestingly, Atg7 deletion in β-cells significantly reduced expression of genes related to UPR (such as C/EPB homologous protein 10, *Chop*; binding immunoglobulin protein, *BiP*; and *Atf4*)^81^. This study demonstrated that inadequate UPR in these Atg7-deficient β-cells have caused cells to be susceptible to ER stressors and increased cell death. Thus, these findings suggest that UPR is an adaptation to protect cells from ER stress rather as a marker of ER stress^81^. Hence, additional research is needed to understand the exact role of Atg7 in β-cells^81^. Another in vivo study with Atg7 deletion specifically in pancreatic β-cells, showed a dramatic reduction in insulin secretion^79^. Thus, autophagy seems to play an important role in β-cell physiology and function^11,79^, yet excessive β-cell autophagy in MetS could be an adaptive response for cell stresses and indication of disease development^83,84^.

The link between autophagy and insulin signaling warrants further investigation to find specific therapeutic targets against MetS-associated metabolic deviations. Proper functioning of the autophagic system is favorable for the insulin signaling cascade; however, when autophagy is disrupted, it induces insulin resistance. Increased levels of mitochondria-derived reactive oxygen species (mtROS) disrupts insulin signaling^85^ and these disruptions could be reversed by removing dysfunctional and aged mitochondria by autophagy to maintain overall cellular homeostasis^23^. Nonetheless, in MetS, increased mTORC1 and reduced FoxO activity (due to hyperinsulinemia) downregulates autophagy in the liver and evokes mtROS production^23,85^. Therefore, insulin-mediated autophagy suppression will ultimately induce insulin resistance during MetS.

## Role of autophagy in heart disease and link to RAS

Autophagy plays a dual role in the cardiac system to maintain a healthy cardiac homeostasis. It can either antagonize or promote disease progression, depending on the context and extent of autophagy induction. In terms of protective role, a plethora of evidence shows that autophagy reduces damage and injuries to cardiac muscle due to cell stresses^86–91^. Under basal condition in cardiomyocytes, autophagy promotes cell survival. However, under cell stress, autophagy gets activated to maintain regular cell structure and function^87^. The functional role of autophagy has been extensively studied at all the stages of heart failure and is only briefly summarized here. A study conducted in patients with dilated cardiomyopathy with fibrosis indicates that the presence of autophagic vacuoles is a potential indicator of heart failure. The authors proposed that induced autophagic activity may prevent myocardial degeneration^88^. By contrast, loss of autophagic activity (Atg5-deficiency) led to heart failure causing cardiac hypertrophy and cardiomyopathy in adult mice confirming the importance of constitutive autophagy to retain metabolic homeostasis in heart^87^. The critical role of autophagy is illustrated in its function to restrict accumulation of misfolded proteins, oxidative stress, and mitochondrial dysfunction during chronic ischemic remodeling. An elegant study by Sadoshima’s group demonstrated that autophagy is activated in cardiomyocytes as a protective mechanism during myocardial ischemia (reduced blood flow to the heart muscle, the myocardium), primarily via AMPK-independent mechanism (inactivating mTOR)^92^. By contrast, during reperfusion (damage caused on tissue, by the blood supply after a period of ischemia) autophagy is activated by Beclin 1 but not by AMPK^92^. Interestingly, apoptosis was also upregulated along with autophagy in reperfusion and when Beclin 1 was inactivated, both autophagy and cardiomyocyte cell death were significantly reduced^92^. Further, during pathological cardiac hypertrophy, excessive autophagy promotes pathological remodeling via accumulation of lipid peroxidation-derived aldehydes. This further documents the discordant roles of autophagy that are tissue and disease-dependent^93^. These results suggest that the functional significance of autophagy may differ at different stages of heart failure and activation of autophagy is not always beneficial^92–95^. Yet, it is not clear whether autophagy is a sign of a repair mechanism in a failing heart or suicidal process for failing cardiomyocytes.

Moreover, irregularities of autophagy are partly responsible for development of atherosclerosis, cardiomyopathy, and hypertension^96–98^. Autophagy-mediated increased proliferation and remodeling of pulmonary artery smooth muscle cells has been identified as a major reason for hypertension^96,97^. This, in turn, narrows the vascular lumen leading to high blood pressure in the pulmonary artery^96,97^. Studies using chloroquine, an antimalarial and antirheumatoid drug which inhibits autophagy, have been shown to reduce pulmonary hypertension in treated subjects^99^. Moreover, autophagy inhibitors or deletion of autophagy gene LC3-II can reduce blood pressure in hypertensive rats^100^, suggesting the participation of autophagy in hypertension mechanisms^99,100^.

**Fig. 6:**
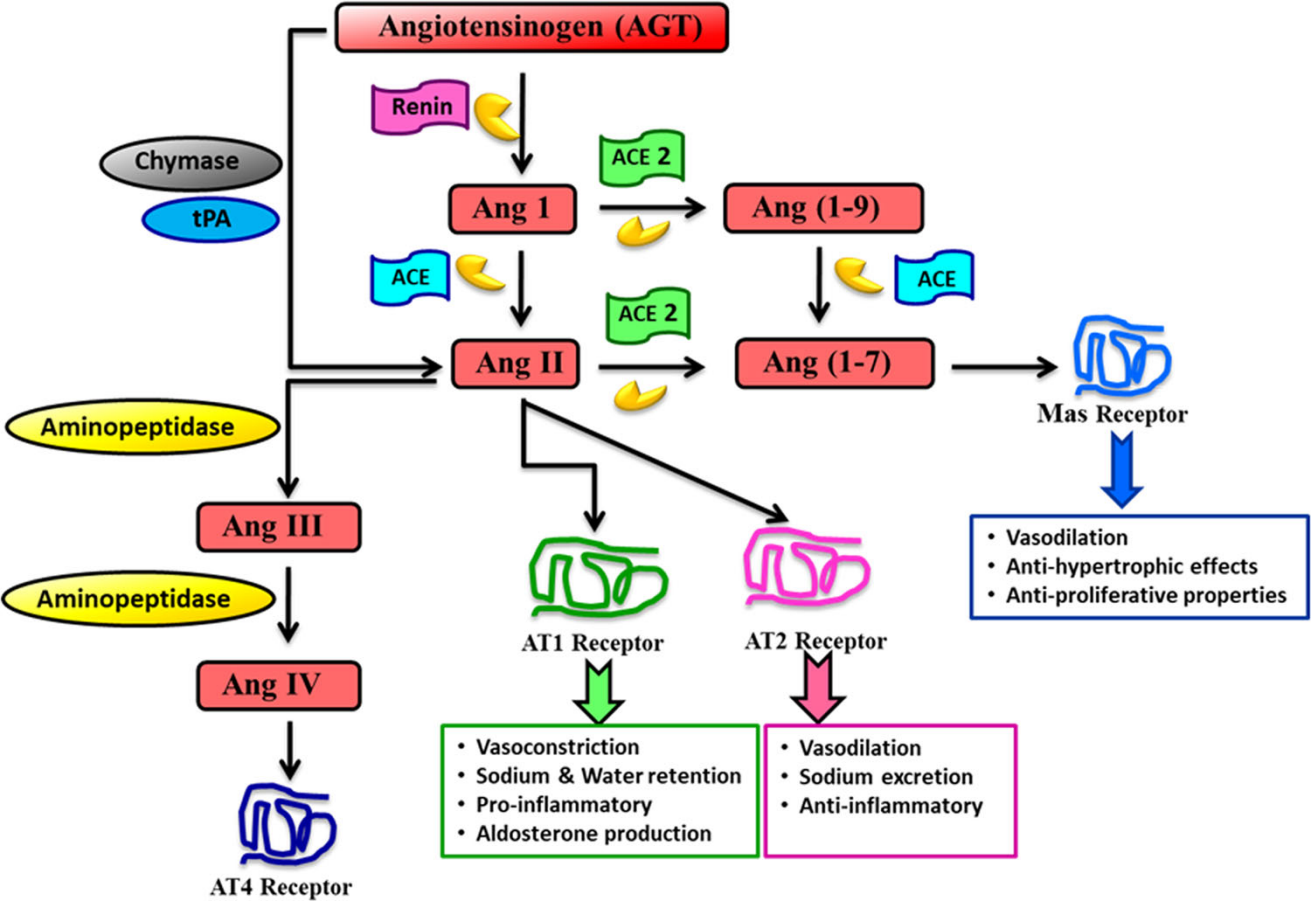
Renin–angiotensin system. Starting component angiotensinogen undergoes several enzymatic cleavages to produce RAS components. Receptors of Ang II, Ang 1–7 along with some of their known functions and specific inhibitors are shown. (Ang: angiotensin, ACE: angiotensin converting enzyme).

Vascular tone is a key component that interferes in blood pressure^101^. It has been suggested that impaired relaxation of smooth muscle cells involving blood vessels may contribute to the pathophysiology of hypertension^102^. Many factors are responsible for hypertension genesis. For example, salt ingestion and angiotensin II (Ang II) are important contributors to high blood pressure^103,104^. Ang II is a major component of RAS. RAS is classically known to regulate blood pressure, electrolyte homeostasis, and fluid balance through its systemic effects^13,105^. The system is comprised of angiotensinogen (Agt), which is the starting component and is cleaved by renin enzyme to produce angiotensin I (Ang I), and angiotensin converting enzyme (ACE) converts Ang I to Ang II (Fig. 6). Agt is converted to Ang II through alternative pathways involving the chymase and tissue plasminogen activator which bypasses the ACE pathway^106,107^. Both Ang I and Ang II are cleaved by ACE2 to produce Ang (1–9) and Ang (1–7), respectively. Ang (1–9) is further converted by ACE to produce Ang (1–7) which then exerts its activities through the Mas receptor^13,16,107,108^ (Fig. 6). The functions of Ang II, the key component of RAS, are mediated through two receptors: Ang II receptor type 1 (AT1) and Ang II receptor type 2 (AT2). Their respective functions are described in detail elsewhere^108,109^. Functions of AT2 are similar to those of the Mas receptor (e.g., vasodilation)^110,111^. Moreover, recent research indicates that aminopeptidases cleave Ang II to produce Ang III and Ang IV, where the end product Ang IV exerts its functions via a newly identified receptor AT4^107,108^. RAS components and their respective receptor interactions have been major clinical targets for decades, and RAS antagonists, such as ACE inhibitors (e.g., captopril, enalapril) and AT1 blockers (e.g., telmisartan, losartan), have been used in clinical studies to control hypertension^112,113^.

Although RAS is known to play a systemic role, components of RAS have been identified as playing a local role in tissues such as adipose, liver, kidney, hypothalamus, and adrenalin glands^16,108^. RAS specifically overexpressed in adipose tissue is involved in obesity, inflammation, ER stress, and insulin resistance^16,109,114^. AT1 and AT2 are expressed in adipose tissue (adipocytes, macrophages, and endothelial cells) and growing evidence confirms the involvement of Ang II in adipocyte growth, differentiation, lipid metabolism, production of inflammatory cytokines, and insulin resistance^16,108,109^. RAS overactivation also activates the nuclear factor kappa B (NF-κB) pathway, which then induces pro-inflammatory cytokines^108^. In addition, Ang II upregulates glycerol-3-phosphate dehydrogenase, a major lipogenic enzyme in adipocytes, leading to accumulation of triglycerides in the adipose tissue resulting in adipose hypertrophy. This is associated with infiltration of pro-inflammatory M1 macrophages, which activate more pro-inflammatory cytokines, creating a vicious cycle^16,108^. Mice overexpressing Agt in adipose tissue have higher pro-inflammatory cytokines and lower anti-inflammatory cytokines expression^108^. Corroborating with this, mice supplemented with ACE inhibitor show 80% reduction in monocyte chemoattractant protein 1 (MCP-1) and interleukin-6 (IL-6) levels^115^. In addition, Agt knockout mice (Agt^−/−^) show reduced blood pressure and reduced adipose tissue growth and lower pro-inflammatory markers^116,117^. In the skeletal muscle, Ang II alters insulin signaling, abolishing insulin-mediated tyrosine phosphorylation of IRS1, and impairing glucose uptake^108^. These results indicate that Ang II activates multiple downstream cascades to induce obesity and MetS, but how these pathways are intertwined needs elucidation.

In vivo and in vitro studies conducted with cardiomyocytes, podocytes, endothelial cells, and neuronal cells show a positive relationship between Ang II (key component of classical RAS pathway) and autophagy^118–124^. Both autophagic activity and Ang II are highly upregulated in the context of cardiac hypertrophy and heart failure^96,125^. Several lines of evidence suggest, RAS-activated autophagy plays a protective role to maintain proper cardiovascular functions at basal and early stages for cardiovascular disease^121,124^. Transgenic (Tg) rat model overexpressing Agt and renin with caloric restrictions exhibited significantly reduced Ang II-induced cardiac damage, cardiomyocyte hypertrophy, and vascular inflammation by activating autophagy^126^. In addition, autophagy protects cardiovascular system by alleviating Ang II-induced oxidative stress and cardiac inflammation^127^. However, prolonged activation of autophagy due to RAS overexpression (increased Ang II and activation of its downstream signaling) could be detrimental^128^. Cardiac hypertrophy caused by Ang II-mediated autophagy in neonatal rat ventricular cardiomyocytes was partially reversed with simvastatin (hydroxymethylglutaryl coenzyme A reductase inhibitor) by inhibiting excessive autophagy^128^. During heart failure, ischemia reperfusion injury was promoted by Ang II^129,130^ and it is further characterized by autophagy-induced cardiomyocyte apoptosis^92^. The underlying mechanisms of autophagy‐induced cell apoptosis is relatively new^131,132^ and additional research in cardiomyocytes with RAS overactivation is warranted to better understand exact molecular mechanisms linking autophagy in the heart to RAS induction.

**Fig. 7:**
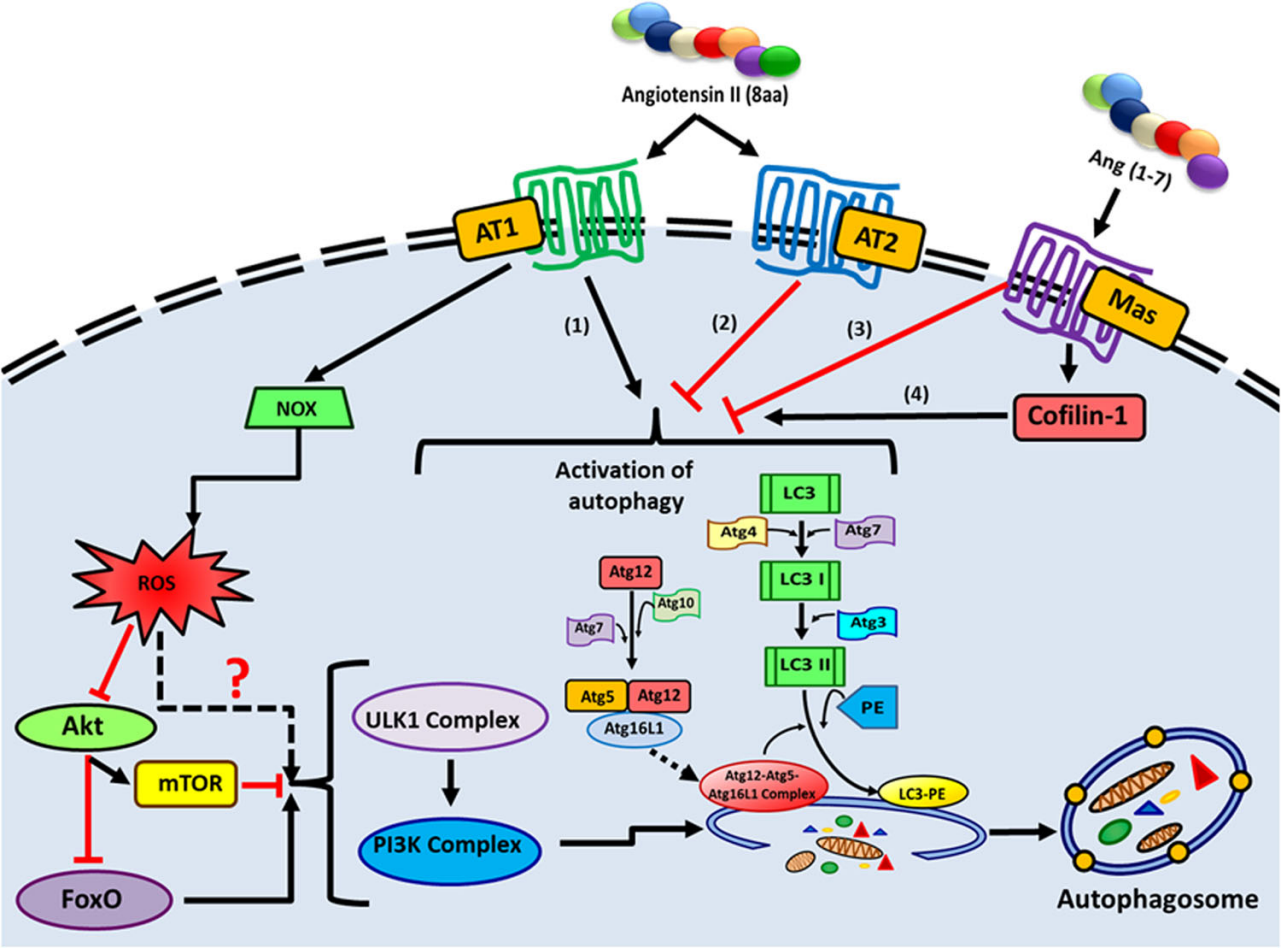
The relationship of autophagy and RAS. Autophagic activity seems to upregulate when RAS is overexpressed. The key player of RAS (1) Ang II induces autophagy via AT1 receptor whereas (2) the interaction of Ang II with AT2 receptor attenuates autophagic function in cardiomyocytes and in neuronal cells. In addition, (3) Ang 1–7 provides a protective function by downregulating autophagy. However, (4) some evidence shows that Ang 1–7 does not inhibit autophagy but induces it via induced cofilin-1 expression. The role of Ang (1–7) on autophagy is controversial and more research is required to understand the underlining mechanisms. Upregulation of autophagy due to RAS overexpression could be via NOX and ROS production. Nevertheless, the underlying mechanisms are yet to be identified.

In vivo studies conducted by Porrello et al. have provided the first evidence of RAS involvement in autophagy in cardiomyocytes. They demonstrated that Ang II increases autophagosome formation significantly when AT1 receptor is highly expressed, and these responses were antagonized by co-expression of AT2 receptor^125,133^ (Fig. 7). Furthermore, Ang II-induced autophagy is completely eliminated by AT1 receptor antagonist losartan. At the same time, AT2 receptor blocker PD1223319 was unable to abolish autophagy, confirming Ang II induces autophagy via AT1 receptor^134^.

Ang 1–7 is another component of RAS (alternative pathway to provide a protective/opposite function) and its role in cellular functions was extensively studied during the past few years^107^. Unlike Ang II, which is positively associated with autophagy, Ang 1–7 shows an inverse relationship with autophagy. Experimental data reveal that Ang 1–7 provides a protective function by reducing oxidative stress and autophagy-stimulated atherogenic cardiac remolding in cardiomyocytes^119^. Ang 1–7 also prevents hypertension-induced excessive autophagic activation in neuronal cells^122^. Rats treated with Ang 1–7 infusion have reduced levels of autophagic markers such as LC3-II and Beclin 1, with increased p62 expression in the brain tissue^122^. Similarly, the antiautophagic role of Ang 1–7 in cardiomyocytes was recently shown by Lin et al. The expression of autophagic makers (e.g., LC3-II) was significantly induced by Ang II, whereas Ang 1–7 reduced Ang II-stimulated autophagy, hypertrophy, and oxidative stress, and provided a protective function in cardiomyocytes^119^. In addition, antagonists of the Mas receptor suppressed the antiautophagy activity of Ang 1–7^119,122^. Interestingly, another study reported opposite results related to Ang 1–7, where Ang 1–7 did not inhibit autophagy, but induced it via cofilin-1 expression in human aortic endothelial cells (HAECs)^135^ (Fig. 7). One probable reason could be that the cells were starved to induce autophagy: it is not clear whether Ang 1–7 treatment caused an additional stress on cells to induce autophagic activity. Nevertheless, the role of RAS components is different depending on the cell type. Therefore, more studies are required to properly understand the molecular mechanisms by which RAS interacts with autophagy in disease development and progression.

**Fig. 8:**
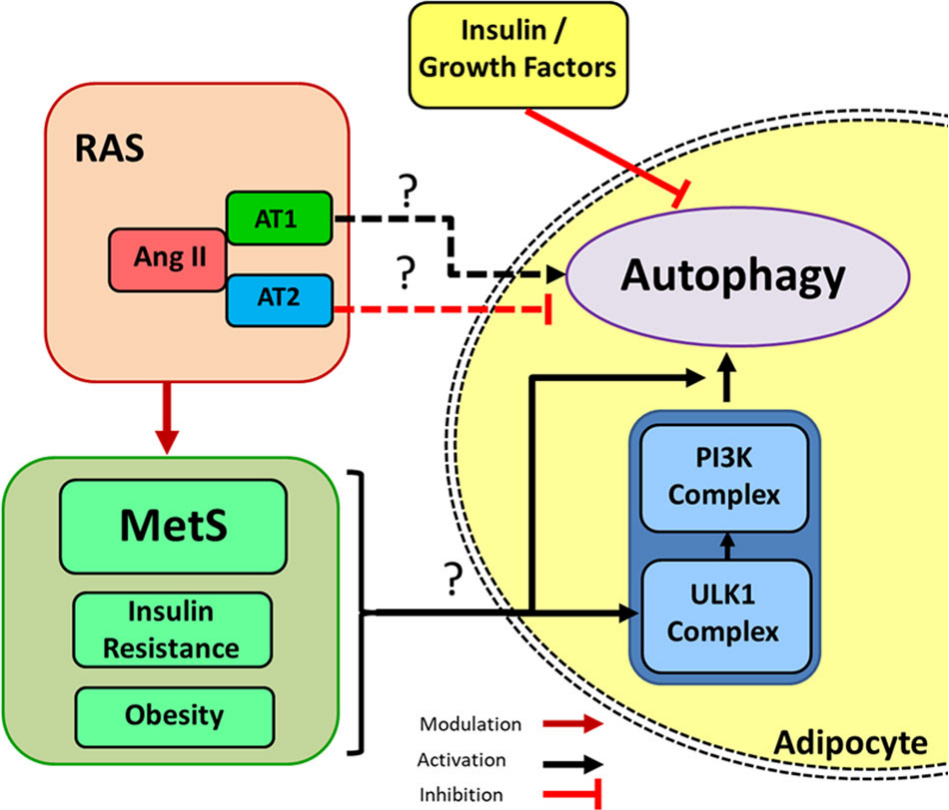
Adipose RAS, autophagy cross talk in metabolic syndrome. RAS is a pivotal system known for controlling blood pressure through angiotensin II and two receptors namely angiotensin II receptor (AT) type 1 (AT1) and angiotensin II receptor type 2 (AT2). RAS overexpressed in adipose tissue may activate autophagy, but the complete role is still unknown.

The interrelationship of RAS and autophagy, specifically in the adipose tissue, is not well studied (Fig. 8). The role of RAS on autophagy in the adipose tissue could be explained by the pro-inflammatory function of Ang II, and RAS-associated cell stresses (oxidative stress, ER stress). Evidence generated from past experiments in adipocytes indicates that Ang II induces Nicotinamide Adenine Dinucleotide Phosphate oxidase (NADPH oxidase) activity, which increases the production of ROS^16^. Induced ROS production, along with pro-inflammatory pathways due to RAS overexpression, could be a potential trigger of autophagy (Fig. 7). ROS could have a direct inhibitory effect on PI3K/Akt pathways (these pathways attenuate autophagy either by activating mTOR or inhibiting FoxO expression) and may involve overactivation of autophagy (Fig. 7). Also, signals related to autophagy initiation (NADPH oxidase activation) may be exerted via AT1 receptor, which is similar to cardiomyocytes or endothelial cells. A study conducted in mouse podocytes by Yadav et al. showed that Ang II induces autophagy through ROS generation. Antioxidants were able to inhibit Ang II-induced autophagosome formation in these podocytes, confirming the involvement of ROS in autophagy when RAS is activated^136^. Nonetheless, these mechanisms need to be explored in adipose tissue to get a clear picture of the contribution of angiotensins in autophagy (Fig. 8).

The effects of Ang II on the mTOR known autophagy inhibitor are interesting. Research conducted in bovine aortic endothelial cells show that Ang II activates mTOR/p70S6K and phosphorylate serine of IRS1, and reduces insulin-induced vasodilation^137^. RAS also increases cardiomyocyte hypertrophy and renal fibrosis via mTOR activation^138,139^. However, whether RAS-activated mTOR is involved in autophagy downregulation is yet to be analyzed extensively, as several in vivo, in vitro studies have demonstrated otherwise (RAS activation is involved in autophagy induction rather inhibition)^120,133,136^. RAS-associated autophagy seems to be regulated by cell stresses including ROS; however, RAS-mTOR interaction in terms of autophagy regulation needs further research since mTOR plays a key role in regulating autophagy in tissues such as liver, muscle, and adipose^38,40,57,140^.

## Association of autophagy and inflammatory phenotype in MetS

WAT has been considered as the main lipid storage organ and also as a key endocrine organ in the human body^141^. It is accepted that obesity is associated with a chronic, low-grade inflammation where macrophages infiltrate into adipose tissue and disrupt adipose function^142^. This is in turn increases higher secretion of pro-inflammatory cytokines^4^ where these cytokines are directly involved in the induction of MetS and other chronic diseases such as cardiovascular disease, Crohn’s disease, cystic fibrosis, and diabetes and it is possible that autophagy is involved in this process as a mediator of inflammatory signals^8,16,17,20,143,144^. The role of autophagy in the secretion of pro-inflammatory cytokines, growth, development, and survival of inflammatory cells (e.g., macrophages, neutrophils, T and B lymphocytes) is important. Some of the well-known pro-inflammatory cytokines regulated by autophagy are IL-1α, IL-1β, and IL-18. When autophagy is disrupted, there is induced secretion of IL-1α, IL-1β, and IL-18 (pro-inflammatory cytokines)^145–148^. In 2008, Saitoh et al. tested the ability of autophagy to reduce pro-inflammatory signaling, showing that Atg16L1 deficiency significantly induces IL-1β production in knockout mice, compared with the controls^149^. Deletion of Atg16L1, Atg7, and Beclin 1 in macrophages increased secretion of IL-1β through TLR^145,146,150^. Nevertheless, several others have shown opposing role of autophagy in IL-1β secretion^151,152^. When autophagy is induced, secretion of IL-1β increases following inflammasome activation^152^. Later, Zhang et al. have further demonstrated that autophagosomes are involved in secreting IL-1β to the cell surface, further confirming a positive interaction of autophagy with IL-1β secretion^151^. In addition, pro-inflammatory cytokines such as tumor necrosis factor alpha (TNFα) and IL-6 are induced by autophagy, thus confirming its discordant role. Evidence from experiments where autophagy is suppressed by treating human peripheral blood mononuclear cells with 3-methyladenine (a mTOR activator by inhibiting PI3K), indicated lower levels of pro-inflammatory cytokines like TNFα and IL-6^145,146,153^.

On the other hand, under homeostatic conditions, pro-inflammatory cytokines also regulate autophagy to provide protection and maintain cell functions^20,154^. Pro-inflammatory cytokines participate in the induction of autophagy^145^: these include interferon gamma (IFN-γ), IL-6, IL-2, TNFα, and transforming growth factor beta (TGFβ). These functions are mediated via NOD-like receptors, TLR, and related signals^145,155^. Autophagy which is induced by pro-inflammatory cytokines plays an important protective role. However, uncontrolled upregulation of autophagy due to over activated pro-inflammatory signals could be harmful as happens in obesity. Higher amounts of IFN-γ in the adipose tissue during MetS induces macrophage infiltration and autophagy in adipocytes^156^. Furthermore, IFN-γ-induced autophagy is also regulated by TNFα. This is based on studies where reduced autophagosome formation and maturation were observed with TNFα blockers^145,153^. It is a chain of events where TNFα-associated upregulation of autophagy (by inducing ROS production) is mediated by activating the Jun kinase pathway and inhibiting the Akt pathway^145,157^. Similarly, in vitro experiments conducted in fibrotic kidney and hepatoma cells have confirmed that another inflammatory cytokine, TGFβ, also upregulates autophagic genes such as Beclin 1, Atg5, and Atg7^145,158^. The involvement of IL-6 in autophagy and tumor progression has been revealed by a recent microRNA study in glioblastoma cells, which demonstrated that IL-6 has an ability to induce autophagy through STAT3 and microRNA-155-3p^159^. Moreover, the pro-inflammatory transcription factor NF-κB has been shown to be involved in the induction of autophagy in response to production of ROS in New Zealand white rabbits: this further confirms the possible involvement of pro-inflammatory cytokines in the activation of autophagic process^160^. In contrast, cytokines such as IL-10, IL-4, IL-13^145,161^ and inducible nitric oxide synthase (iNOS)^162^ have autophagy-suppressing ability. This repression is possibly mediated via activating the Akt pathway (normally involved in inhibiting starvation-induced autophagy), and signal transducer and activator of transcription 6 (STAT6) signaling^145,161^. IL-4 and IL-13 affect the later phase of autophagy process, which prevents autophagosome-lysosomal fusion to inhibit autophagy^161^.

## Discussion and final remarks

Autophagy is involved in regulating gene and protein expression and acting as a key regulator of several intricate cascades. Various mechanisms have been proposed to explain the link between autophagy and MetS: adipocyte differentiation, lipid metabolism in hepatocytes, protective or detrimental role in cardiomyocytes, pancreatic β-cell maturation, endothelial cell function, and others. Our discussion brings new insights into the pathway that could lead to better pharmacological treatment for MetS and develop targets for alternative treatments.

Autophagy is linked to obesity by reducing the number of mitochondria in adipocytes. This in turn, lowers β-oxidation of lipids and increases fat accumulation rather than oxidation. The complexity of autophagy also increases due to its modulation by aging^163^. In contrast to higher accumulation of lipid droplets in the livers of adult mice due to autophagy suppression (e.g., Atg7 deletion), young Atg7-knockout mice displayed lower hepatic lipid buildup and triglyceride amounts^163^. These results indicate that autophagy may be a key regulator of early hepatic lipid metabolism and is involved in the maintenance of energy metabolism. This is seen during adipocyte differentiation^36,37^ (Tables 1 and 2), but the pattern of autophagy-related genes regulation may be different in adulthood. In a study by Pyo et al. using Atg5 Tg mice to stimulate autophagy, ubiquitous Atg5 overexpression increased lifespan and reduced cell stresses^164^ (Table 1). There was no significant change in the body weight among young Atg5 Tg and control mice. After 12 months, however, 12% average weight reduction was observed in the Atg5 Tg mice compared to the control. In contrast to what Pyo et al. described, mouse studies conducted with systemic or tissue-specific Atg5 or Atg7 knockouts in adipose tissue^9,36,42^ or muscle^29^ have shown reduced body weight and resistance to diet-induced obesity, along with other manifestations of MetS. This indicates that inhibition of autophagy, but not its stimulation, has beneficial effects under certain experimental conditions. This controversy can be attributed to several reasons. Autophagy gene knockouts are often lethal at the embryonic stage or after a few days from birth (e.g., Beclin 1, Atg7, and Atg5 knockouts) when gene deletion is performed ubiquitously^45,56,165^. This confirms the importance of autophagy genes in the early ontogenesis, whereas reduced body weight in Atg-knockouts can be explained by impaired development. Second, when gene (Atg5 and Atg7) deletion is restricted to the adipose tissue or muscle, reduced body weight and improved health conditions (e.g., insulin sensitivity, fat loss) were observed^9,29,36^. However, it was harmful when the gene was deleted in the liver^56^.

It is beneficial to inactivate autophagy specifically in adipocytes, yet in the long run, continuous inhibition of autophagy may lead to impaired adipose function including defective adipocyte differentiation, insulin resistance, abnormal cytokine production, and cell stresses (ER and oxidative stress) due to accumulation of cytoplasmic wastage, such as unfolded proteins and damaged organelles (e.g., mitochondria) within the cells. Therefore, permanent blockage of adipose autophagy may not always be beneficial, but temporary inhibition by adipose-specific drugs during the early stages of obesity would be ideal to minimize adverse effects of activated autophagy. Further, adipocytes of the knockout mice models (which were generated using adipose-specific promoters) are affected during their early development stages. Hence, whether autophagy inhibition in fully matured adipocytes (later stages of adipose differentiation or during obesity), would provide similar beneficial effects as seen in these mouse models remains to be elucidated. Similarly, stimulation of autophagy in one place, for instance, liver, probably would not compensate for the adverse effects associated with autophagy activation at the same time in other organs: for example, in the adipose tissue, which is associated with obesity, insulin resistance, and MetS induction.

Other messenger molecules may also be involved in the regulation of autophagy-associated functions and their effects may differ depending on the cell type and tissue. Although the changes in expression of autophagy genes and proteins could confirm the involvement of autophagy in obesity, it may not be the only indicator of autophagy flux. However, results from different tissues (adipose, muscle, and liver) shed light on the biological significance of autophagy and the use of autophagic genes as potential therapeutic targets to prevent obesity. Taken together, these results indicate that many different roles of autophagy: in the liver, for example, autophagy may have a protective function for lipotoxicity by preventing fat accumulation and retention, whereas in the adipose tissue, autophagy promotes adipocyte growth for better energy storage. Yet these actions could be regarded as complementary in order to maintain metabolic homeostasis.

“Globesity” as coined by the World Health Organization is an important public health problem and frequently associated with diabetes and hypertension. Although high blood pressure is linked to MetS, the exact mechanisms by which autophagy is involved in MetS remain unraveled. It is plausible that unique key regulators may mediate the deleterious effects of autophagy as well as other cellular functions. Since AT receptors are expressed in adipose tissue, RAS plays a vital role in autophagy mediation; however, all these studies were focused on a paucity of cell types including cardiomyocytes, podocytes, endothelial, and neuron cells. Therefore, further experiments in different cell lines and tissues (e.g., adipose, liver and muscle) are required to better understand the role of angiotensins in autophagy. Interestingly, there were no studies directly linking or addressing the significance of RAS in autophagy in adipose tissue. Since adipose tissue plays a crucial role in MetS, it is critically important to understand the relationship of RAS and autophagy and its underlying mechanisms. Addressing these knowledge gaps sooner rather than later is imperative for future development of therapeutics to prevent MetS and associated diseases.

Autophagy pathway modulation by ACE inhibitors, such as enalapril (largely used as antihypertensive drug^166,167^) remains obscure. Some studies demonstrated that ACE inhibitors may reduce MCP-1, IL-6 and might restrain the pro-inflammatory phenotype of patients with MetS. Moreover, the AT1 blockers, known as “sartans”, (e.g., Losartan) are widely prescribed for hypertension and seem to influence autophagy. In a study performed with cancer cell lines using fimasartan, an intriguing type of cell death mediated by autophagy was amplified, while the metastatic capacity of cancer cells was reduced^168^. Although in some cell types a positive relationship has been suggested between RAS and autophagy, it is still unclear whether blocking RAS would modulate autophagy. As discussed above, Ang II promotes autophagy through AT1 and reduces autophagy activity by engagement with AT2.

In closing, overall, autophagy plays a protective role in the body, yet it can have a substantially harmful role in obesity, MetS, and aging. Thus, both stimulation and inhibition of autophagy could be beneficial, depending on other confounding factors and disease conditions. The ambiguous role of autophagy among different tissues and how it can be exploited to facilitate MetS treatment remains undiscovered and merits further investigations. We suggest that the RAS-linked autophagy pathway might be a key contributor to MetS. Thus, unraveling the RAS pathway and how it controls autophagy using pharmacological or immune selective therapy may help break the vicious cycle that contributes to MetS burden and establishment.
